# Hypertrophic Perianal Herpes Mimicking Squamous Cell Cancer: A Case Report and a Review of the Literature

**DOI:** 10.7759/cureus.30649

**Published:** 2022-10-24

**Authors:** Shreshtha Singh, Harshdeep Tyagi, Archana Khanduri, Nalini Bansal, Rahul Gupta

**Affiliations:** 1 Gastrointestinal Surgery, Synergy Institute of Medical Sciences, Dehradun, IND; 2 Anaesthesiology, Synergy Institute of Medical Sciences, Dehradun, IND; 3 Pathology, Shree Guru Gobind Singh Tricentenary (SGT) University, Gurugram, IND

**Keywords:** cutaneous squamous cell carcinoma (scc), immunosuppresion, azathioprine treatment, autoimmune pancreatitis (aip), herpes simplex

## Abstract

Clinical practice frequently involves the discovery of perineal lesions. The human papillomavirus, molluscum contagiosum, and herpes simplex virus are to blame for the majority of these anogenital lesions. In the majority of cases, these lesions may be identified by their distinctive appearance. It is challenging to make a clinical diagnosis in immunocompromised people since these lesions might be large and have uncommon appearances. Verrucous perianal herpes is a rare type of herpes that resembles squamous cell carcinoma in gross appearance. We present a case of a 71-year-old man on azathioprine, an immunosuppressive drug for autoimmune pancreatitis, who developed a perianal lesion resembling squamous cell carcinoma. Excisional biopsy revealed a benign ulcerative lesion with herpetic inclusions. The patient received antiviral treatment, and the perianal wound completely healed. He developed a similar lesion in the perineum at one year follow up, which was successfully treated with oral and topical antivirals.

## Introduction

Anogenital lesions are most frequently caused by herpes simplex virus (HSV) infections worldwide [1]. Superficial painful ulcers are a hallmark of the ulcerative form of anogenital herpes. It is the most frequent clinical manifestation in immunocompetent people [2]. However, in immunocompromised patients, the clinical presentation may be unusual with the emergence of large ulcers and superadded bacterial infections. Occasionally, genital herpes may appear in a pseudo-tumoral hypertrophic form that mimics squamous cell carcinoma (SCC) [3]. A tissue biopsy is necessary in these circumstances to provide a definitive diagnosis [4]. Here, we discuss a case of a perianal lesion masquerading as SCC in an elderly male on azathioprine for autoimmune pancreatitis. Excisional biopsy revealed a benign ulcer with herpetic inclusions. The patient was successfully treated with antiviral treatment for primary and recurrent perianal lesions in the follow-up period.

## Case presentation

**Figure 1:**
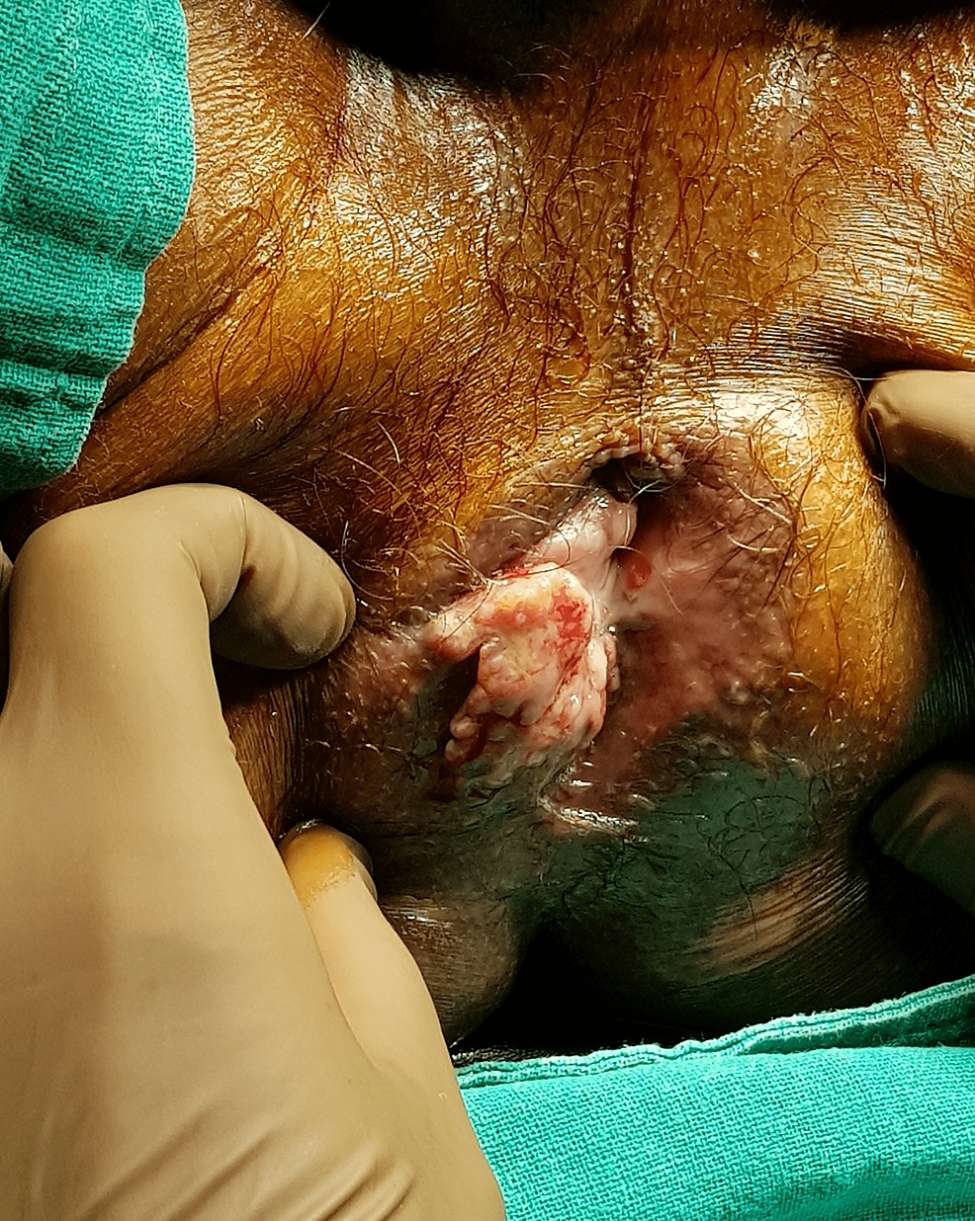
Perianal herpes at the site of hemorrhoidectomy mimicking squamous cell carcinoma

A 71-year-old male presented with perianal lesion with discharge for seven months. He disclaimed having fever, urinary issues, or constipation. He had a history of surgical excision of histologically proven prolapsed hemorrhoids a year prior to the presentation. For autoimmune pancreatitis, he had been on azathioprine 50 mg/day for two years. He had recurrent self-limiting herpes labialis five years back. There was no history of any other systemic illness, such as diabetes mellitus. On perianal examination, a firm, non-tender, well-defined lesion measuring 3 x 4 cm was present at the prior hemorrhoidectomy site (Figure 1).

The lesion had a variegated appearance and an uneven surface. No inguinal lymphadenopathy existed. Abdominal examination was unremarkable. His human immunodeficiency virus (HIV) test was negative. Due to irregular margins, variegated appearance, and location at a previous scar site, squamous cell carcinoma was suspected, and an excisional biopsy was planned. Under spinal anesthesia, a wide local excision of the perianal lesion was performed and sent for microscopic examination. Recovery from surgery was uneventful. 

**Figure 2:**
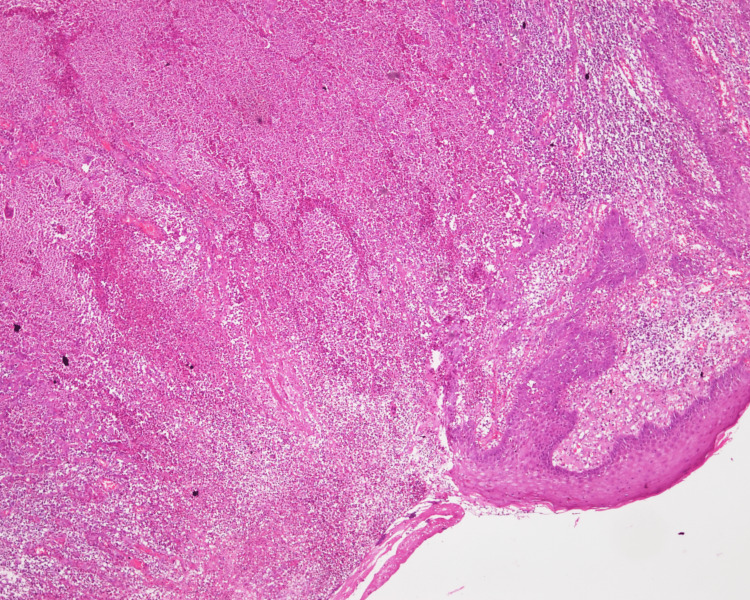
Microscopic appearance of the edge of the ulcerative lesion showing pseudo-epitheliomatous hyperplasia and dense inflammatory infiltrates (H&E, 20x)

Histological analysis revealed tissue bordered by epidermis with an ulcer coated in thick neutrophil exudates (Figure 2).

**Figure 3:**
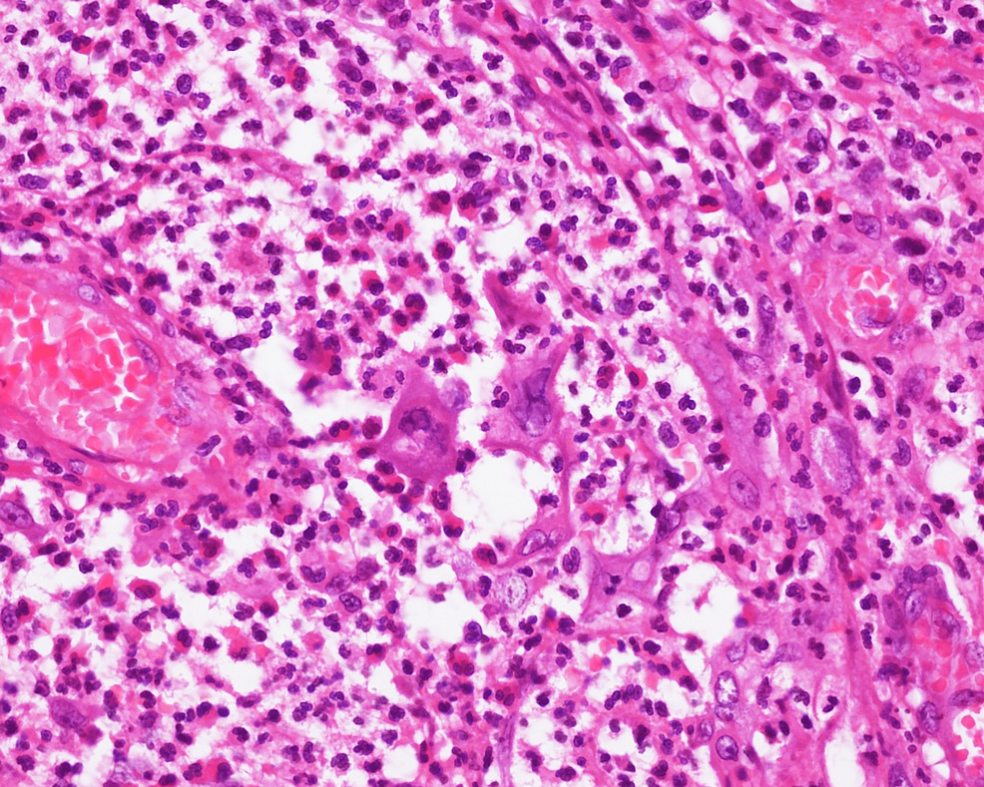
Microscopic examination of the perianal lesion showing dense infiltration with neutrophils and plasma cells with occasional multinucleated keratinocytes (H&E, 40x)

Infiltration of plasma cells was present along with pseudo-epitheliomatous hyperplasia (Figure 3).

**Figure 4:**
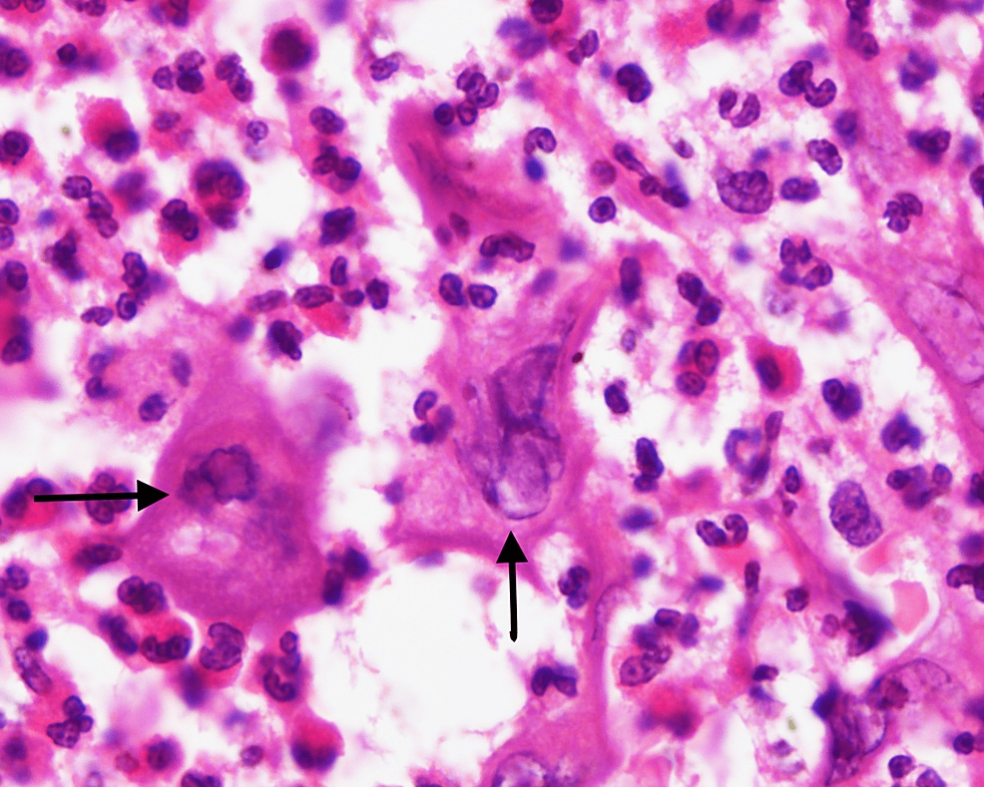
High-power microscopy revealed the keratinocytes with multinucleation, ground-glass nuclei, and chromatin margination, suggestive of viral inclusions (arrows) (H&E, 100x)

The keratinocytes at the ulcer's edge displayed viral inclusions in the form of ground-glass nuclei, multinucleation, and chromatin margination that suggested herpetic infection (Figure 4). Additionally, sporadic intranuclear Cowdry A inclusions were discovered. Malignant cells weren't seen.

The patient received a dermatology consultation and was administered acyclovir (5 mg/kg/day) for 14 days. On the follow-up visits, the perianal wound healed in six weeks, and the patient continued to be on azathioprine. One year after the primary surgery, he presented with a similar perineal lesion with foul-smelling discharge at another site. The new lesion was successfully treated with oral acyclovir and topical imiquimod. However, azathioprine therapy was not stopped considering the risk of recurrent acute pancreatitis in the future after consultation with a gastroenterologist.

## Discussion

The most common cause of anogenital verrucous lesions is due to human papillomavirus (HPV) infection [5]. However, anogenital infections can occur due to varicella-zoster virus, cytomegalovirus (CMV), molluscum contagiosum, and herpes simplex virus (HSV). Anogenital lesions are frequently observed in immunocompromised patients, especially those with HIV infection [6]. But, there is no correlation between the degree of immunosuppression and the risk of developing anogenital lesions. Moreover, it is unclear why this hypertrophic variant of HSV infection is most frequently found in patients coinfected with HIV. The possible immunological mechanisms for the development of hypertrophic lesions include the production of tumor necrosis factor (TNF)-alpha by an increased number of factor XIII-positive plasmacytoid dendritic cells, promotion of the keratinocyte growth rate and resulting acanthosis and hyperkeratosis. Moreover, decreased IFN-gamma production, which plays an important regulating role in keratinocyte activity, may also help to explain the evolution into the hypertrophic form [7].

HSV comes in two varieties: HSV-1, which is frequently linked to oropharyngeal infection, and HSV-2, which is primarily linked to genital illness. However, in affluent nations, HSV-1 has recently emerged as a frequent cause of anogenital herpes, most likely as a result of rising orogenital sex usage. HSV is transmitted by contact with the mucosal surfaces, vaginal secretion, or oral secretion of an infected person. Unusual methods of transmission include fomites and aerosols. In immunocompromised and homosexual males, HSV1 and HSV2 typically attack the anogenital squamous epithelium and manifest as genital or perianal ulcers and blisters. Less frequently, tumor-like nodules or condylomatous lesions can result from anogenital HSV infections [6]. In immunocompromised patients, HSV can also affect the rectum, nasal cavity, bronchial system, and eyes. HIV individuals have been reported to have the majority of HSV's unusual manifestations. However, there is a substantial risk of HSV infections with unusual presentations in patients on immunosuppressive medications. Rectal lesions in HIV patients can have a wide range of pathogenic origins, including lymphogranuloma venereum, syphilis, and CMV [8]. It's also crucial to take non-infectious diseases like lymphoma and squamous cell carcinoma into account in this population. Most of the HSV cases in immunocompromised patients are due to reactivation of the latent virus than primary HSV infection, as seen in the present case [9].

The presence of exophytic and painful tumours with well-defined boundaries and ulcerated surfaces, seen in the perianal region, vulva, penis, and scrotum, clinically defines HSV lesions [10]. Similar lesions, though, have also been reported in extra-genital regions. Based on a comparison of clinical and history data, HSV isolation, and the exclusion of other infectious causes, the diagnosis is made. The Tzanck smear test is a useful bedside test to detect herpetic infections. Multinucleated giant cells can be seen in up to 60-70% of cases [10]. Other simple tests such as wet mount, 10% potassium hydroxide mount, tissue smear, Gram's stain, and Venereal Disease Research Laboratory (VDRL) test should be performed to rule out other sexually transmitted diseases. Variable epidermal hyperplasia can be seen histologically, along with multinucleate epithelial cells and a dense mixed dermal inflammatory infiltration consisting of lymphocytes, plasmacytes, and eosinophils. Since small samples may be insufficient for diagnosis and obscured by the strong inflammatory response to the virus, an incisional skin biopsy is advised. Viral culture, polymerase chain reaction (PCR), and immunofluorescence staining are a few of the techniques that can be used to diagnose HSV. Immunohistochemistry has emerged as one of the most effective methods among them, particularly when dealing with uncultivable microorganisms [11]. Using certain monoclonal antibodies, this extremely sensitive and precise method can distinguish between a variety of viruses, including HSV-1 and HSV-2 [11]. Due to cost constraints, these tests were not done in the present case.

Azathioprine is one of the most widely used immunosuppressants for various illnesses, such as inflammatory bowel disease, autoimmune diseases, and post-transplant organ rejection. Although it is not a very strong immunosuppressant, cases of atypical HSV and herpes zoster infections have been reported in patients on azathioprine therapy [12,13]. A prospective study by Seksik et al. also reported a significantly increased risk of HSV infections with azathioprine in inflammatory bowel disease (IBD) patients [14]. One of the possible mechanisms is immunological derangement in natural killer (NK) cells and lymphocytes [12]. 

Most HSV infections are self-limiting in immunocompetent individuals. However, in immunocompromised patients, HSV infections should be treated with antiviral drugs. First-line systemic antiviral medications, such as acyclovir (oral and intravenous), valacyclovir, and cidofovir, have been utilized, though with varying degrees of success. However, hypertrophic genital herpes is frequently resistant to these medications. Imiquimod is a topical immunomodulator that has anticancer and antiviral properties, as well as the ability to synthesize and release a number of endogenous pro-inflammatory cytokines, including interferon-alpha. Although there are no randomized studies, topical imiquimod has been shown to be effective in treating persistent genital herpes in immunocompromised patients [15]. It was also found to be effective in treating hypertrophic HSV infection resistant to standard medical care [16]. Additionally, immunosuppressants may have to be stopped, changed, or the doses need to be reduced to treat HSV infection and prevent recurrence [14]. 

## Conclusions

Immunocompromised individuals, especially those receiving immunosuppressants such as azathioprine, are prone to develop anogenital herpes. HSV infections with an atypical presentation should be taken into consideration as a possible differential diagnosis while dealing with perianal lesions in immunocompromised patients. There should be a low threshold for biopsy in suspicious cases to prevent misdiagnosis. Treatment of HSV infections includes systemic and topical antiviral therapies, cessation of immunosuppressants if possible and surgical excision of localized disease if refractory to medical therapy.
